# Experiences With Wearable Activity Data During Self-Care by Chronic Heart Patients: Qualitative Study

**DOI:** 10.2196/15873

**Published:** 2020-07-20

**Authors:** Tariq Osman Andersen, Henriette Langstrup, Stine Lomborg

**Affiliations:** 1 Department of Computer Science University of Copenhagen Copenhagen Denmark; 2 Department of Public Health University of Copenhagen Copenhagen Denmark; 3 Department of Communication University of Copenhagen Copenhagen Denmark

**Keywords:** consumer health information, wearable electronic devices, self-care, chronic illness, patient experiences

## Abstract

**Background:**

Most commercial activity trackers are developed as consumer devices and not as clinical devices. The aim is to monitor and motivate sport activities, healthy living, and similar wellness purposes, and the devices are not designed to support care management in a clinical context. There are great expectations for using wearable sensor devices in health care settings, and the separate realms of wellness tracking and disease self-monitoring are increasingly becoming blurred. However, patients’ experiences with activity tracking technologies designed for use outside the clinical context have received little academic attention.

**Objective:**

This study aimed to contribute to understanding how patients with a chronic disease experience activity data from consumer self-tracking devices related to self-care and their chronic illness. Our research question was: “How do patients with heart disease experience activity data in relation to self-care and chronic illness?”

**Methods:**

We conducted a qualitative interview study with patients with chronic heart disease (n=27) who had an implanted cardioverter-defibrillator. Patients were invited to wear a FitBit Alta HR wearable activity tracker for 3-12 months and provide their perspectives on their experiences with step, sleep, and heart rate data. The average age was 57.2 years (25 men and 2 women), and patients used the tracker for 4-49 weeks (mean 26.1 weeks). Semistructured interviews (n=66) were conducted with patients 2–3 times and were analyzed iteratively in workshops using thematic analysis and abductive reasoning logic.

**Results:**

Of the 27 patients, 18 related the heart rate, sleep, and step count data directly to their heart disease. Wearable activity trackers actualized patients’ experiences across 3 dimensions with a spectrum of contrasting experiences: (1) knowing, which spanned gaining insight and evoking doubts; (2) feeling, which spanned being reassured and becoming anxious; and (3) evaluating, which spanned promoting improvements and exposing failure.

**Conclusions:**

Patients’ experiences could reside more on one end of the spectrum, could reside across all 3 dimensions, or could combine contrasting positions and even move across the spectrum over time. Activity data from wearable devices may be a resource for self-care; however, the data may simultaneously constrain and create uncertainty, fear, and anxiety. By showing how patients experience self-tracking data across dimensions of knowing, feeling, and evaluating, we point toward the richness and complexity of these data experiences in the context of chronic illness and self-care.

## Introduction

### Consumer Wearable Activity Trackers in Chronic Care

Consumer health information technologies such as wearable activity trackers are increasingly being considered to improve chronic care management [1-4]. Contrary to traditional health information technologies, these devices are developed as consumer devices and not as clinical devices. Most commercial activity trackers aim to monitor and motivate sport activities, healthy living, and similar wellness purposes. Wristbands such as Fitbit and smart watches that track bodily signs (eg, heart rate) do not provide diagnostic services or disorder-specific information, and they are regulated less rigorously than are monitoring devices aimed at specific patient groups and clinical measures. This makes them readily available to consumers. Moreover, their designs aim at easy and noninvasive integration into everyday life, by way of automated tracking. These features are attractive for application in chronic disease management, where a healthy lifestyle can be a central part of treatment, rehabilitation, and prevention [1,5-8].

The separate realms of “wellness tracking” and “disease self-monitoring” and “activity data” and “medical data” are thus blurred, which is somewhat mirrored in an increasing prominence of concepts such as “patient-generated health data” and “personal health technology” where the focus is on the individual producer of data, rather than on the specific context or purpose of use. Applying leisure activity tracking to chronic care management provides new opportunities but is often based on assumptions about what characterizes these devices: easy, applicable, user-friendly, empowering, and motivating technology that can collect data with relevance for self-care and treatment. While there are great expectations and promising results emerging [2,3,9], little attention has been paid to the embodied and embedded experiences of self-tracking among patients with a chronic disease using consumer devices, which are not integrated in the health care system.

In this paper, we explored how patients who use consumer wearable devices make sense of them and of the data they produce and display vis-à-vis their embodied disorders. We recruited 27 patients with chronic heart disease who had an implantable cardioverter-defibrillator (ICD) to wear a Fitbit wearable tracker to understand the experiential qualities of how they relate self-tracking and activity data to their disease to explore the question: How do the embodied experiences and self-care practices of dealing with a specific health condition respond to the introduction of activity trackers?

### Background and Significance: Experiences with Self-Monitoring in Health Care and Leisure Contexts

The existing literature of experiences with self-tracking comprises two related fields: (1) rich literature on how patients, as part of their prescribed treatment, engage in and make sense of clinically validated data related to self-care for chronic illnesses and (2) emerging literature that primarily explores users’ experiences with leisurely oriented self-tracking technologies that are outside of the health care system. Patients’ experiences with consumer activity-tracking technologies designed for use outside the clinical context have received scant academic attention. There is an important research gap in understanding the relation between the rich human-information interaction and the contexts of activity tracking such as self-care [1,10]. Consequently, studies that explore the experiences of people coping with illnesses — while recognizing the specific ramifications that self-tracking might have for those who have severe health problems — are necessary.

### Self-Care and Chronic Illness in eHealth

Many patients routinely engage with data from digital devices that are part of the prescribed treatment. These clinically integrated data-producing devices affect self-care activities as they enable managing symptoms, taking medicine, dealing with the emotional impact, and tackling lifestyle changes [11-13]. Fostering self-care has been a central ambition of much telecare and electronic health (eHealth) development during the last 20 years. Early telecare technologies were typically designed from a clinical standpoint with measures to support remote decision making such as blood glucose tracking in diabetes [14,15], oxygen saturation, pulse rate and respiration rate tracking in chronic obstructive pulmonary disease [16], and heart arrhythmia detection through remote monitoring of cardiac implantable electronic devices such as pacemakers and ICDs [17]. In recent years, a more participatory agenda frames eHealth innovation, aiming to enhance independence and enable patients to become more active participants in managing their own disorder (eg, through use of wearable activity trackers) [18,19].

While much research has focused on measurable outcomes of digital self-tracking and self-management on clinical parameters, emergent studies have also explored the experiential qualities of patients’ “data work” [20-22]. Central to such data work, along with the experiential qualities of patients engaging with self-tracking data in chronic care management, are the affective aspects. It is known that self-monitoring of blood glucose data by patients with diabetes and their caretakers is tightly bound to an emotional struggle ranging between control and freedom, peace of mind and anxiety, and empowerment and the burden of managing technology [23]. Similarly, it is found that self-tracking data in fertility self-monitoring promotes the achievement of certain positive goals but may accentuate negative emotions such as feeling burdened or abandoned [24]. Others have studied patient experiences in heart arrhythmia telemonitoring and found that not having access to data or feedback from clinicians has an emotional and life-changing impact, which in turn creates doubt, guilt, and concern [25].

### Self-Tracking and Activity Data

While clinically validated self-monitoring technologies have become the standard in chronic care, the rise of low-cost sensors has accelerated the consumer market, making wearable activity trackers and mobile data-logging applications a widespread commodity. Large corporations like FitBit and Apple are entering the medical domain with consumer wearable devices, most prominent through automated activity measures like step count [1,26] to support disease monitoring and rehabilitation of cardiac, pulmonary, and cancer patients [8,27-29], among others, and most recently, through large-scale interventions using the Apple Watch for screening of atrial fibrillation.

Literature on the so-called Quantified Self over the past decade has explored users’ (ie, self-trackers) experiences with these technologies and the data they produce when applied voluntarily for leisure or wellness activities [30-34]. Lomborg and Frandsen [35] showed that, while self-tracking is often depicted as an entirely individual endeavor to retrieve calculable, reliable knowledge, it is experienced by users as a deeply communicative activity, with an often playful and pleasurable quality to it.

In their exploration of self-tracking cycling, Lupton et al [36] presented the concept of “data sense” to describe how people’s experience with data from sensors is not just a matter of cognitive “knowing” — as often assumed in data literacy approaches — but equally involves sensory and affective dimensions such as alerting cyclists to new bodily sensations while possibly invoking feelings of frustration or even embarrassment. Thus, what emerges are accounts of self-tracking experiences as complex encounters between the metric and sensuous, between knowing and feeling. This, in turn, suggests that self-tracking, while often a purposeful and systematic practice, is not necessarily guided by goals of improving the self, forming new and healthier habits, or getting to know oneself better, as suggested by many of the wellness technologies currently entering the market [37].

The critical question is then: what happens when technologies, practices, and data from the consumer market for self-tracking are introduced in the context of chronic self-care? What kinds of experiences do they offer in a setting where self-tracking is “pushed” [38] to patient-consumers who live with a chronic disorder?

Several studies have examined patients’ experiences with wearable activity trackers, and the focus tends to be on patient acceptability and feasibility among the elderly [39-41] and in the chronic care context [1,5-8,28]. Some support positive outcomes like ease-of-use and willingness among patients to wear activity trackers and integrating them into clinical care [5,28]. Rosenberg et al [28] conducted a 3-week study examining the acceptability of the Fitbit Zip and attitudes towards integrating fitness tracking into clinical care among men with prostate cancer. All participants were willing to wear the device and endorsed its value in ensuring they engaged in a “minimal amount of activity.” However, several barriers to use were found, including health-related limitations (like pain and injuries making it difficult to walk) and practical or technical problems with syncing devices and experiencing data inaccuracies (eg, not capturing the activities).

Other studies present negative aspects and challenges with integrating patient-generated data in clinical settings [6,7]. Zhu et al [6] found technical challenges (such as security and privacy issues and the practical work of clinicians transferring self-tracking data to the electronic medical record), social challenges (such as health professionals adapting to new forms of care where patients are collaborative partners), and organizational challenges (like organizational policies and workflows that do not include attending to patient-self tracking data). Ancker et al [7] conducted an interview study to explore self-tracking among patients with multiple chronic conditions. They found that patients associated several negative experiences with self-tracking and self-tracking data can negatively influence the patient-clinician relationship owing to a lack of trust in the data. For these patients, self-tracking thus became burdensome, which contrasts the pleasurable and playful experiences promoted in wellness self-tracking.

### Objective

The objective of this study was to understand how patients with chronic heart disease, as opposed to healthy individuals, experience activity data from consumer self-tracking devices when engaging in self-care. With this study, we contribute to the emergent literature on how patients’ experiences with consumer wearable activity trackers are related to their illness and their self-care activities and the implications that arise for design and deployment of these devices. There is a need for going beyond acceptability and feasibility studies and conducting more fine-grained analysis of the experiential qualities of interacting with personal health data outside the context of clinical practice among the increasing number of people with chronic illnesses.

## Methods

### Overview

We conducted a qualitative study to understand how patients with an ICD experience self-tracking of activity data in relation to their embodied condition and daily practices of dealing with a chronic heart condition. As we know from the self-tracking literature, such experiences may change over time [33]. To grasp this, we followed patients over a 49-week period from January 2018 to December 2018, during which we observed their activity tracking data and interviewed them repeatedly (2-3 times each) about their experiences and insights gleaned from the data.

### Setting

This study was part of a larger research and development project, SCAUT (Self-, Collaborative- and AUTo-detection of signs and symptoms of deterioration), 2014–2018, which aimed to improve early detection of deterioration and communication among patients with a cardiac device and health professionals. The overall project was carried out at a cardiac device clinic at the Rigshospital, University of Copenhagen, Denmark, which is one of the largest cardiac device remote monitoring centers in Europe, following more than 3500 patients.

### Recruitment of Participants

The study comprised a sample of 27 patients with chronic heart disease who had a secondary prevention ICD and were already part of an (R&D project). Secondary prevention ICDs were offered to individuals who survived sudden cardiac arrest or had a history of dangerous and recurrent abnormal heart rhythms, which is relevant for this study due to the chronicity of the disease and related self-care activities. While patients were similar in having an ICD, their underlying cardiac diagnosis, possible comorbidities, and psychosocial situation differed substantially. Participants were recruited through a mix of purposive sampling and self-signup to ensure that patients were interested and not too ill to participate. Of the 65 ICD patients we invited to participate, 27 ICD patients provided written informed consent to this study, which explored the experiences of activity data as related to being an ICD patient and the data’s potential for predictive analytics of dangerous arrhythmias. Of the 27 patients, 25 patients were male (93%), and 2 patients were female (7%); the average age was 57 years. The sample largely reflected the demographic profile of ICD patients in Denmark in 2017, when 18% of patients were female and the majority of procedures were carried out on patients aged 55-74 years [42].

The participants were provided with and instructed to wear a Fitbit Alta HR (Fitbit, San Francisco, CA), which is a wristband activity tracker that can record and visualize heart rate, sleep, and steps onscreen and in a Fitbit mobile app. They were informed that wearing the activity tracker was unrelated to their treatment at the clinic and that our purpose was to explore how they experience the relationship between activity data and their heart disease.

### Data Collection: Semi-Structured Interviews

Data were collected with 66 semistructured interviews in 3 overall iterations using 3 interview guides. The first iteration aimed to create a baseline of patients’ expectations concerning activity-illness relationships and get a sense of their embodied experience of everyday living with an ICD. The second iteration aimed to understand the initial 2-week experiences with activity tracking using concrete examples from their own tracking data and asking them what they had learned or wondered about when using the Fitbit and looking at the data. The third iteration aimed to understand the longer-term experiences and data-sensing practices and any ambivalences arising from using the Fitbit (4–49 weeks).

Patients were interviewed individually (sometimes with relatives) in their homes or in locations convenient to them (eg, workplace or hospital office space). Each interview lasted between 25 minutes and 1 hour and 40 minutes. During interviews, the interviewers took field notes and pictures of participants showing concrete examples of activity data in the Fitbit mobile app to support the analysis of the data. All interviews were audio-recorded and transcribed either in full or in selected passages.

### Data Analysis

The interview transcripts were hand-coded iteratively, following an abductive reasoning logic [43,44], starting with a joint analysis workshop after the second interview with all 27 patients to identify emergent themes regarding data sensing and insights from the data to be followed up in the final interviews. At this stage, field notes and pictures from the interviews offered an interpretive aid, offering contextual guidance for those of us who had not been present at the interview. Upon the final round of interviews, another joint workshop solidified and elaborated the initial insights and ideas against the body of related work, to develop a joint analytical framework and coding protocol. This framework combined dimensions of data sensing — knowing, feeling, and evaluating the self through data with contextual embedding and experiences of illness in daily chronic living [45] — to clarify how patients with an ICD make sense of Fitbit data relating to their heart disease. Finally, we recoded the complete empirical material manually according to these dimensions, first individually and then together, to ensure reliability in producing a thematically organized analysis of patients’ data sensing [46]. 

### Study Approval and Ethical Considerations

This paper was based on a substudy of the SCAUT research and development project, which was approved by the Danish Data Protection Agency and reviewed by the National Board of Health and Danish National Committee on Health Research Ethics (H-19029475). We took several measures to respond to possible ethical concerns. First, we ensured voluntary participation through an open invitation with self-signup, and we emphasized in interviews that participants could opt out at any time. Second, we communicated with all participants between interviews to ensure they were comfortable with wearing the wristband. We made sure that the participants understood that the Fitbit activity tracker was a consumer device and not a clinical device and that the data did not have diagnostic validity. Finally, we adjusted the conversation in interviews accordingly if patients expressed specific health concerns brought on by their interaction with the Fitbit; specifically, we urged them to contact their health professional for guidance.

## Results

### Device Engagement

Our results showed how patients with an implanted ICD device engaged with and made sense of activity data from the Fitbit in the context of chronic illness and self-care. Most (18 of the 27 participants) related their real-time heart rate, sleep, and step count data directly to their heart disease (Table 1). The remaining participants, however, connected the data to leisure activities, wellness, and exercise. Some portrayed themselves as not being a patient, explaining that they mostly did not have symptoms. Two patients chose to opt out after only wearing the tracker a few times owing to finding the wristband annoying to wear or simply losing interest (P6, P7). Patients used the activity tracker for an average of 26.1 weeks and took breaks from using it an average of 5.9 weeks.

The patients who did relate the data directly to their illness did so in 3 overall dimensions (Table 2): as something that generated new knowledge, as something that raised affective responses, and as something that could be used to evaluate themselves and their overall health. Within these 3 dimensions of experience, patients accounted for a range of positive, negative, and ambivalent experiences with activity data. For an extended analysis of the affective dimension and the consequences for patients’ interpretation of Fitbit data, see [47].

**Table 1:** Overview of participating patients with chronic heart disease and an implantable cardioverter-defibrillator (n=27).

Patient number	Age (years)	Sex	Year of ICD^a^ implant	Symptoms experienced	Number of weeks using the Fitbit (not using)	Experienced Fitbit data relating to heart disease
P1	67	Male	1998	No symptom experiences; experienced palpitations before	18 (0)	No
P2	61	Male	2015	Severe chest pain and shortness of breath	47.5 (0.5)	Yes
P3	41	Male	2009	No symptom experiences; experienced shortness of breath before	41 (6)	No
P4	55	Male	2014	Dizziness and sometimes fainting	13 (5)	Yes
P5	66	Male	2010	Dizziness and sometimes fainting	8 (23)	Yes
P6	67	Male	2015	No symptom experiences	9.5 (1.5)	N/A^b^
P7	28	Male	2008	No symptom experiences	6 (3)	N/A
P8	69	Male	2015	No symptom experiences (primary prophylaxis)	36.5 (11)	Yes
P9	47	Male	2008	No symptoms experiences related to his heart disease; lung disease; difficulties exercising	33,5 (14.5)	Yes
P10	61	Male	2010	Sometimes feeling very tired	30 (8)	Yes
P11	59	Male	2006	Shortness of breath and sometimes sleep problems; finds it difficult to feel his heart rate	8.5 (0.5)	Yes
P12	66	Male	2015	Dizziness and sometimes fainting; anxious about getting a shock and experiences depression	49 (0)	Yes
P13	67	Male	2017	No symptom experiences; rarely experience fainting; leg tenderness and muscle fatigue	44.5 (0.5)	Yes
P14	52	Female	2008	No symptom experiences	49 (0)	No
P15	61	Female	2004	No symptom experiences; sometimes being anxious about the ICD/irregular heartbeats	14 (1)	Yes
P16	47	Male	2014	Dizziness and sometimes fainting	35.5 (11.5)	No
P17	45	Male	2013	Dizziness and shortness of breath during high activity levels	35 (7)	Yes
P18	67	Male	2009	No symptom experiences	18 (29)	Yes
P19	66	Male	2005	Palpitations daily; experiences periods of depression and has restless leg syndrome	20.5 (1.5)	No
P20	69	Male	2014	No symptom experiences, except sometimes shortness of breath	39.5 (8.5)	Yes
P21	38	Male	2008	No symptom experiences; worries daily about having a cardiac arrest again	13.5 (0)	Yes
P22	59	Male	2001	Shortness of breath and sometimes dizziness when exercising and running; rapid heartbeats and chest pain	19.5 (0.5)	Yes
P23	49	Male	2017	No symptom experiences	47 (2)	Yes
P24	74	Male	2017	No symptom experiences	42.5 (6)	Yes
P25	51	Male	2014	No symptom experiences	9 (15)	No
P26	56	Male	2010	No symptom experiences; sometimes feels palpitations and shortness of breath	4 (0)	No
P27	58	Male	2014	Shortness of breath; sometimes sleep problems; knee-pain due to osteoarthritis	12 (5)	Yes

^a^ICD: implantable cardioverter-defibrillator.

^b^N/A: not available because the patient did not describe a relation between the activity data and his or her heart disease.

**Table 2:** Dimensions of how patients experienced activity tracking related to their disease.

Experiential dimension		Experience	
**Knowing**			
	Positive: gaining insight		Learning that heart disease increases one’s average resting heart rate (P2, P4)
			Learning that medication influences the heart rate (P5, P22, P23, P27)
			Learning that activity improves one’s average heart rate (P4, P21)
			Using activity data to monitor heart pumping ability (P10)
	Negative: evoking doubts		No new learnings: Sensing is more useful than activity data (P1, P5, P16, P22)
			Doubting heart rate data (P2, P22)
			When doubt becomes mistrust (P12)
**Feeling**			
	Positive: being reassured		Feeling safe through Fitbit reassurance (P11, P12, P17)
			Reassurance prompts activity (P24)
	Negative: becoming anxious		Both insights and doubts can introduce new anxieties (P12, P13, P15, P23)
**Evaluating**			
	Positive: promoting improvement		Being nudged and getting praise (P19, P20, P23, P24)
	Negative: exposing failure		Recognizing a nudge but not knowing what to do about it (P13)
			Not getting the proper reward: the invisibility of “good” activities (P18)
			Self-disappointment with poor numbers (P8, P17, P24)
			Ignoring or resisting nudges (P18, P19)

### Knowing: Gaining Insight and Evoking Doubts

As part of their motivation for participating in the study, many participants expressed an interest in knowing more about their health and body and about the possible relationships between their daily activities and their heart. In our interviews, it became clear that patients actively sought knowledge about their heart and health through the readings on the Fitbit and that some gained insight from the data. For others, the knowledge they gained from sensing their own bodies was more useful than the Fitbit data. Finally, some participants experienced that the data did not align with their activities and sensory experiences; thus, they doubted the accuracy and trustworthiness of the data.

#### Learning That Heart Disease Increases One’s Average Resting Heart Rate

One patient gained insight into his average resting heart rate and discovered it was higher than he expected:

My resting heart rate ought to be 60, but it is 80, and as soon as I start moving, it goes up to 100 or 120.

P2

It did not concern him since there is not much the clinicians can do about it, as he said. Another patient had the same kind of insight:

Then, the pulse is in a zone where I am physically active. That is what it signals. But I am not physically active. I am just walking. That has been an eye-opening experience.

P4

#### Learning That Medication Influences the Heart Rate

Several patients knew, speculated, or learned about how medication can affect the heart rate. By looking at the Fitbit data, P5 noticed that his average heart rate decreased when on vacation, and he considered that his medication might have an effect. P27 also noticed that his medication influenced his heart rate:

Obviously, the heart rate follows how active you are; but, for healthy people, it’s not abnormal that it reaches 116. But I get 13 pills in the morning and 3 in the afternoon.

P23 also noticed that medication influences his heart rate:

My beta blockers have been reduced in dose, which may also have something to do with the changes in the resting heart rate.

P22 had always been exercising; however, after an event a few years ago, he was prescribed a double dosage of beta-blockers, and it was decided to reduce the dosage again because his heart rate had dropped too low. Now, he finds it exciting to use Fitbit to learn about the relationship between medication, exercise, and heart rate and to confirm his heart rate data:

I still monitor what happens. This morning I saw an increase in my average heart rate of 12% and my maximum heart rate of just over 15%. And that's what’s fun and what I use it for because it has annoyed me extremely that I couldn't run like I used to.

#### Learning That Activity Improves One’s Average Heart Rate

P21 noticed that there is a relationship between exercising and average heart rate:

After serious ice hockey training, the average heart rate is higher on the day after, and then it drops in the weekend.

Similarly, P4 discovered that when he is more active, his average heart rate drops:

During the 20 days I have been wearing the Fitbit, I have seen a drop in my heart rate by 10 heartbeats per minute. I think that’s crazy. I have been more active, yes, and I have been walking more. I have set exercise goals.

He experienced this as a positive thing:

I think it’s fine that my resting heart rate goes down because, if it goes down, the heart does not have to work as hard. It makes a difference whether my heart rate is 70 or 60 beats per minute when resting.

#### Using Activity Data to Monitor Heart Pumping Ability

Several patients had experience with other activity trackers. One patient (P10) described how he used the data to monitor his heart condition. He was diagnosed with heart failure, and his heart had a reduced pumping ability, “Down to around half of what is normal,” he said. He used the Fitbit to address his concern that his heart rate will drop even lower:

I use it to keep an eye on my condition. When I go spinning, I do the same intervals, and I can therefore see if I have burned the same calories in that hour. So, I can say, okay, I'm fairly stable, or I can see if it has gone down. Because then it might be my heart getting worse in its ability to pump.

#### No New Learnings: Sensing is More Useful Than Activity Data

Several patients had learned over the years to sense developments in their heart condition. One patient explained that, in the past, when he got rapid and dangerous heartbeats and the ICD began treatment with antitachycardia pacing to terminate the arrhythmia, he sat down and waited until it passed. He used to call the Heart Centre when he felt the symptoms to get confirmation about the episodes. For him, the Fitbit did not generate any new insights apart from what he already knew from sensing and listening to his body when exercising in the gym:

I know I cannot do physically very demanding exercises. I have come to terms with that. So, I have not received any new extra information via Fitbit.

P1

P22 found it useful to use the activity tracker to get confirmation on fast heartbeats, for example, when running in the woods. However, he trusts his senses more:

If there is a connection between what I feel in my body and what the tracker shows, then I react. But when the tracker shows something that I don’t notice in my body, I consider it an IT error.

Another patient explained that he has tried to look at the Fitbit data when he gets sudden dizziness; however, it provides no explanation:

Sometimes I suddenly experience a “dive,” and then I just have to hold on to something. It’s very different how often it happens. But when I look at [the Fitbit activity data], it does not show anything. So, I can’t find the reason for getting so dizzy.

P5

Similarly, P16 explained that there is no connection between symptoms and heart rate:

There is no connection. I've tried to get symptoms when doing gymnastics where I had the pulse all the way up, and I've tried to get symptoms while I was sleeping. I have not found any connection at all.

#### Doubting Heart Rate Data

Heart rate data also created doubt among some participants. One patient doubted that the heart rate data presented on his Fitbit were accurate when walking:

But I have seen sometimes when out for a walk, my heart rate is really high. Average 166—I think that is a little high. And, sometimes, I have seen it going up to 197 beats, and I don’t know why.

P2

Another patient found the heart rate data “weird” at times. He experienced chest pain a few times when he was out running; however, no answers could be found in the activity data:

I felt a little uncomfortable — one might suddenly think of a blood clot. But I can't see in any of the trackers that the pulse has been particularly high or particularly low.

P22

At other times, when he was just walking, there were sudden peaks in the heart rate data:

It seems strange. It's just right there — a peak up. It almost seems like a mistake that it goes from 80 to almost 170. It seems completely messed up what happens here.

P22

Several other patients experienced that their heart rate showed unusual fluctuations on Fitbit.

#### When Doubt Becomes Mistrust

One patient had disease-related anxiety and had difficulties sleeping. Initially, the Fitbit data concerning his sleep revealed that he slept more than he thought he did when he woke in the morning after a difficult night. However, when he later saw that the Fitbit had registered “sleep” while he was calmly watching a movie, he and his wife started distrusting the measurements of sleep altogether:

This Saturday, we looked at sleep, for instance, and we could not make the numbers fit because he had been awake a lot. The data was wrong, and we do not know how it works. Then you go on to think, can you trust this device at all?

wife of P12

Across participants, the sleep tracking measures of Fitbit were noted as unreliable and not reflective of actual sleep. For some participants, discovering that sleep data were inaccurate led to mistrust and a general, critical understanding of the measurements provided by Fitbit.

### Feeling: Being Reassured and Becoming Anxious

Being diagnosed with a heart condition often introduces profound anxiety into patients’ (and their relatives’) lives. It is well-known that patients with an ICD are at an increased risk of being diagnosed with depression and anxiety [48]. In our interviews, some patients had different levels of anxiety and used the Fitbit to reassure themselves that their heart was doing okay. For some, this helped them engage more in physical activity — something they might have held back on owing to fear of provoking an attack (ie, kinesiophobia) [49]. However, the Fitbit — partly owing to the doubts and uncertainties introduced as described — could also spark new and additional anxieties and negative feelings regarding participants’ health.

#### Feeling Safe Through Fitbit Reassurance

For some patients, having a heart condition raised their embodied attention, making them very alert to bodily signs:

As soon as there is even a little thing in these zones [in his chest region], and I would even say just one, like a sprain, I get nervous.

P12

This patient, who is very affected by anxiety and depression, used the Fitbit to reassure himself that he is not having a cardiac arrest:

Then, I was out chopping firewood. I felt like it began to beat both in a weird way, and it felt like it beat really fast, but it did not. There was nothing. There was nothing to be seen [on the Fitbit] anyway.

P12

For this patient, seeing his heart rate within the normal spectrum reassured and calmed him down:

Now I get certainty. Is something wrong or not.

Another patient, who experienced getting a shock from his ICD after walking the stairs, explained that*:*

Being able to see my heart rate is normal creates a sense of security because I’m not able to feel when my heart rate rises. A normal rhythm means that there is nothing to be afraid of — no danger is underway.

P11

Similarly, P17 used the heart rate data as support in vulnerable situations:

I've tried it so many times, to have those VTs [rapid heartbeat] and I know what it leads to, and that's what I fear.

One time he was lying down, and he used the heart rate on the Fitbit tracker to get confirmation on the duration of rapid heartbeats:

If it lasted more than 5 minutes, I would have called 112 [emergency services]. Because, sometimes, I think when I get that feeling of fast heartbeats, it may well be imagination.

#### Reassurance Prompts Activity

Holding back during physical activity was something several of the informants touched upon, as some had had a heart attack while exercising or had experienced an ICD shock when climbing a flight of stairs. Checking their heart rate while doing more physically demanding tasks and sports motivated them to increase their activity:

I’m not afraid to have a high pulse when we do the Bikefit exercise at gymnastics … because I can see it goes down again [his heart rate].

P24

#### Both Insights and Doubts Can Introduce New Anxieties

In the first section, we touched upon some of the doubts concerning the validity of the Fitbit data. For the anxious patient in need of reassurance, this uncertainty can be stressful, as noted by the wife of P12. P15 described it as follows:

There are plenty of worries when you have a heart disease. You don’t need unnecessary things that make you worry more.

The Fitbit sleep data, for example, created unwanted attention to what it meant for her health:

What does it mean for my health? Am I sleeping enough or too little, and what can I do about it? Such concerns arise, which I could do well without.

She also experienced getting a high pulse that was “completely unprovoked” and then seeing it on the Fitbit:

But I can't do anything about it, and I can't use it for anything—unless they can see it in the clinic.

One patient noticed that Fitbit wants him to sleep 8 hours per night — a goal he rarely reaches:

I have always had this sleep pattern, and it did not bother me until I got this Fitbit, which says I should sleep 8 hours a night … I get worried and start to question whether I ought to sleep more.

P13

Finally, the introduction of new concerns and anxieties by Fitbit goes beyond patients’ individual experiences. P23 explained that he tried to avoid drawing attention to his Fitbit wristband when being around his teenage kids:

I think it reminds them a little bit of something bad … it’s interpreted negatively—like someone has to keep an eye on me.

### Evaluating: Promoting Improvement and Exposing Failure

As we presented in the previous sections, patients used Fitbit’s numerical representations to make sense of their bodily sensations in the context of self-care. The fact that the Fitbit device allows them to see — as numbers, icons, and graphics — something that they usually relate to as sensations provides a new form of motivation. Setting targets for activity, getting notifications, and seeing achievements represented visually can be encouraging. Concurrently, however, the Fitbit also exposes unmet goals, thereby inducing self-disappointment or even shame. The Fitbit does not register or “see” all the activity that the patients found to be relevant as fair representations.

#### Being Nudged and Getting Praise

Several of the patients talked about how the Fitbit nudged them to stay physically active — it is *“a kick in the butt,”* as one patient noted (P19). Users can set their activity goals as they please; however, most went with the default setting of 10,000 steps a day. All patients looked daily to see if they had reached that goal, and some found it motivating to see the numbers and get positive feedback (visually provided in the form of stars) from the user interface. One patient described himself laughingly as:

Addicted to it because it says you have to walk 10,000 steps a day, and that fits with some of our walks. It becomes a sport; it gets me going.

P20

Others noted how the gamification element invoked in the Fitbit led to small changes in their daily activities. For P23, Fitbit prompted him to take the stairs instead of the elevator to get more steps. It also prompted him to get up when it beeped every hour to take 250 steps by walking up and down the hall during work breaks. For these patients, Fitbit is “a little push in the right direction” (P23) and “an inspiration to continue” (P24).

#### Recognizing a Nudge But Not Knowing What to Do About It

Whereas simple nudging features such as awarding stars for accomplishing specific activity benchmarks seemed to motivate participants, there were also examples of participants becoming unsure of what constitutes appropriate activity. For example, Fitbit (by default) beeps once every hour during the day to pace the wearer to walk 250 steps every hour. For P13, this nudge suggested that average but regular activity throughout the day might be preferable to his usual practice of lumping activity together for more intense periods of exercise. It made him wonder if he should organize his workout differently, even if this wondering did not lead him to make any actual change.

#### Not Getting the Proper Reward: The Invisibility of “Good” Activities

After wearing the Fitbit for some time and having acquainted themselves with the collected activity data, some participants reported being frustrated that the Fitbit did not really measure all their activity. They did not feel “seen” by the device and rewarded properly for their efforts. This is particularly the case for those participants who did cycling, CrossFit, and other activities beyond walking and running as part of their everyday life activities. One participant lamented:

It is actually misleading because most of my activity is on a bike, and it does not register this. But it is also exercise.

P18

These experiences added to the doubt in the data and the accuracy of measurements described previously.

#### Self-Disappointment With Poor Numbers

If positive feedback is seen as motivating further activity tracking, conversely, the negative feedback from Fitbit made some participants feel disappointed with or ashamed of themselves because it highlighted that they had not been active enough:

Well, I guess it has to do with that bad conscience you get the next day, if you cheat.

P8

This was mentioned by several patients, and, for some, it made the Fitbit less attractive. P24 talked of self-disappointment when receiving negative feedback from Fitbit and noted that there might be someone else looking at their data, surveilling whether they reached their goals:

Yes, because it gossips all the time, noting that I have not walked far enough … I think you could always find an excuse for not walking; like, it’s raining.

For P17, the low step count created negative emotions on “bad days” and became linked to his heart condition:

It gives me a little guilty conscience that I do not get much exercise because, I have no doubt, the more weight I gain, the more fat is generated around my heart, and the harder it is for the heart to pump.

#### Ignoring or Resisting Nudges

Some participants tempered their engagement with Fitbit by actively resisting to do what the device suggests:

I know that it tells me, every once in a while, that it is time to go for a walk. But I decide when it is time to go for a walk. And then it says, let’s go, and I’m like no way because I don’t have the time right now.

P18

For some, the Fitbit’s nudging was simply a source of annoyance:

It can be a little irritating watching all the green “pling pling.” I don’t want that; I don’t care about it. I just want the info.

P19

Yet, for others, such as P18, resisting the nudge to walk every hour was followed by a deeper reflection about whether activity tracking is good at all for his health:

I actually think it is a little unhealthy to measure oneself all the time. It comes to take up a lot, in my life, and I don’t think it is that important.

## Discussion

### Principal Findings

Our study contributes to understanding how patients with chronic heart disease, as opposed to healthy individuals, experience activity data from consumer self-tracking devices in self-care. We found that patients with an implanted ICD relate the activity data to their illness experience and their self-care activities in 3 overall dimensions: as something that generated new or destabilized existing knowledge, as something that raised affective responses, and as something that could be used to evaluate themselves and their overall health.

The distribution of patients’ experiences on a continuum from positive to negative suggests that activity data had “dual effects,” which means that the data created as much as it solved the problem of chronic illness [50]. The problems that people with chronic illness have to deal with become mediated in new ways and what may count as “normal,” “good,” “problematic,” or “bad” may change accordingly. Positive and negative effects potentially co-exist and support the experiential ambivalence that studies of self-tracking and activity data have also found among leisurely users and quantified self-enthusiasts [22,45,51,52]. The concept of “ambivalence” unites this stream of research in which patients’ attitudes towards digital health devices “neither are consistently negative (implied by the notion of ‘rejection’) nor consistently positive (implied by the notion of ‘acceptance’)” [45]. Conflicting or ambivalent experiences appear constitutive of self-tracking: “doubt, guilt, fear, shame, dismay, disappointment, and hesitation as well as joy, relief, excitement, enthusiasm, and pride” [51].

Generating knowledge from interpreting activity data is often portrayed as the essence of self-tracking. For healthy individuals, it may comprise discoveries about physical performance in everyday life and adopting healthier behavior [33,35]. For the patients in this study, performance-oriented and fitness-oriented development of self-knowledge also surfaced. Patients obtained new insights about how exercising improves their average heart rate and that their heart disease may be the reason for a higher resting heart rate.

Other more disease-specific reflections surfaced as using Fitbit data to monitor the status and development of heart failure (heart pumping ability) and speculating about how heart medication affects the pulse. Unusually high heart rate data created doubt when walking or when connected to chest pain while running. Therefore, Fitbit data became part of generating a type of lay and personal expertise for, at best, supporting day-to-day self-care activities and living with a chronic disease and, at worst, creating uncertainty. This kind of “experiential knowledge” or “patient knowledge” [53-55] is often considered distinct from medical and scientific knowledge in that it is a by-product of bodily sensing and coping with daily practicalities of the disease as well as it is re-appropriated medical knowledge used to contribute, but also dispute, the biomedical perspective [55].

For patients, Fitbit data can provide support for self-care with informational cues alongside bodily sensations and experiences in the development of “know-now” [53] (ie, understanding what is going on or deciding what action to take). As opposed to healthy individuals’ knowledge-making with Fitbit, the unsupported lay interpretation of medically unvalidated heart rate data poses a risk for patients taking inappropriate action, for example, using Fitbit heart rate numbers to diagnose a cardiac arrest when running and deciding whether to keep on running or when getting chest pain and becoming dizzy in the office and then using Fitbit to decide what to do. The practical implications of patient knowledge generation from Fitbit suggest that patients should not be left alone with interpreting activity data as part of self-care. Deploying self-tracking and activity data in chronic care should be carefully accompanied by a purposeful clinical intervention such as rehabilitation and training programs where clinical staff can support patients in interpreting activity data, and data visualization should be designed to support meaningful action in the context of self-care.

The affective dimension of self-tracking when living with a chronic heart disease also emerged as loaded with ambivalence. Fitbit numbers may provide numerical reassurance, which can relieve acute anxiety related to unclear bodily sensations and provide confidence to exercise. Concurrently, heightened attention to Fitbit data can also introduce new uncertainties and anxieties. Moreover, it is important to underline that the reassurance of the Fitbit data is not based on clinical evidence and, while reduction of acute anxiety is important to patients’ wellbeing, there is a risk that the numbers provide pseudoproof not sufficiently reliable to indicate anything clinically relevant about the patient’s condition. Given the prevalence of mental health issues, such as anxiety and depression, related to heart disease and the lack of mental health services for these patients, it is vital to consider the potential negative interactions between health tracking and mental health. Patients with chronic mental health comorbidities should not be left to try to cope with serious mental health issues alone with consumer devices.

Taken together, we see a tension between Fitbit’s promotion of success and exposure of failure to comply with set standard activity levels in the actual experience of using Fitbit. The ambivalence of knowing, feeling, and evaluating one's chronic health condition against activity data from consumer devices, as opposed to clinically validated instruments, poses a concern for how engagement with data is placed in chronic care contexts as well as the purposes inscribed in the design of these new devices. In their analysis of ambivalence in mobile health for HIV care, Marent and colleagues [45] argued for the need to consider how the tension implied with ambivalence is embodied by particular bodily conditions and embedded in particular relationships and environments. Our study concerns people who, in clinical terms, have a chronic condition; however, the embodiment of this condition varies among participants. For some, the disease has a continuous and very challenging presence related to managing and coping with severe symptoms (see Table 1), while others tell us they do not feel sick at all. We suggest that the ambivalence of Fitbit data is more problematic when used by people who have a chronic condition and even more so for people who feel very challenged by their disease.

This relates to a second point about embeddedness. The ambivalence of Fitbit data should be understood in relation to its embeddedness in everyday contexts unrelated to clinical contexts of treatment. To what extent do people in these contexts have support from others such as relatives, peers, or health professionals who choose to engage in handling the ambivalences they encounter? What resources can they mobilize to act when experiencing doubt, anxieties, or other concerns when self-monitoring with Fitbit? With our paper being specifically concerned with patients who engaged with Fitbit data outside the established relationships of health care institutions, these questions become critical. Navigating benefits and harms of this form of active engagement with personal health data is, to a large degree, dependent on individual circumstances, resources, and networks, leaving inequalities potentially less mitigated by public health systems. We find that these are very central insights to take into account in research that focuses on how self-care practices can be furthered by harnessing the power of data and personal health technology. Too often, this literature focuses narrowly on individual information processing and empowerment while disregarding the relational and situational embeddedness of chronic disease management. It not only neglects insights from health information-seeking literature, which has convincingly shown that patients’ information behavior is more often based on serendipity, avoidance, blissful ignorance, indolence, bewilderment, and indolence than on rational choices and reflections [56], but also neglects that self-care practices are always collective in the sense that they are embedded in complex sociomaterial relationships [57,58]. What our study further adds to the literature on self-care and technology, then, is that technology and data mobilized outside the established arrangements of health care with consumer tracking devices may introduce new ambivalences that some patients may have difficulties managing without professional assistance.

For human-computer interaction research and the design of activity-data visualization for patients, our study aligns with the understanding that data supports different levels of reflection and serves multiple purposes [59]. The implications of our study, we suggest, is that consumer activity-tracking devices deployed in health care contexts should be designed to also support collaborative reflection (ie, “co-reflection”) with health professionals, rather than focusing mainly on individual reflection [60]. Research on ways to support the shared work of tracking [61], co-reflection, and “co-care” [62] might be necessary to consider for personal informatics research in chronic self-care [25,47,63].

As an increasing number of people are generating and interacting with digital and individual health data outside the context of clinical practice, issues of inequality in health must be considered. Thus, there are important implications to consider in this typically optimistic, yet blurred, realm of “personal health data” (actualized for wellness purposes) and “patient-generated data” (actualized for clinical purposes).

### Conclusions

We presented the findings from an explorative intervention study of how patients with a heart arrhythmia who have an implanted ICD experience activity data from Fitbit concerning their self-care and chronic illness. The aim was to further emergent literature and offer crucial empirical insight into the introduction of wellness tracking devices to various forms of chronic care management and the associated user experiences.

Through repeated semistructured interviews with 27 patients equipped with a Fitbit wristband, we offer support and further elaboration on existing work on patients’ ambivalent experiences. We found that wearable activity trackers actualize patients’ experiences across 3 dimensions on a spectrum: (1) gaining new knowledge versus evoking doubt, (2) feeling reassured versus becoming anxious, and (3) evaluating one’s health by celebrating improvements and exposing failure. The experiences of individual patients can reside more on one end of the spectrum, can reside across all 3 dimensions, or can combine contrasting positions and even move across the spectrum over time. While activity data from wearable devices may be a resource for self-care through reassurance and motivation, they may also constrain patients and create increased uncertainty, fear, and anxiety.

The ramifications of knowing, feeling, and evaluating one’s chronic health condition against activity data from consumer devices, as opposed to clinically validated instruments, are largely unexplored. Our study suggests that we need critical attention in scholarship and health care practice concerning how engagement with such data is practiced in chronic care contexts, not least to assess how the purposes inscribed in the design of these new devices may be molded and twisted in self-care when meeting the logics, needs, and abilities of patients in different health care circumstances. Designers and health authorities should consider this complexity and ambiguity when determining the usefulness of self-tracking data in chronic illness.

## Abbreviations

eHealth: electronic health
ICD: implantable cardioverter-defibrillator
